# Cross-species analysis of apical asparagine-rich protein of *Plasmodium vivax* and *Plasmodium knowlesi*

**DOI:** 10.1038/s41598-018-23728-1

**Published:** 2018-04-10

**Authors:** Fauzi Muh, Md Atique Ahmed, Jin-Hee Han, Myat Htut Nyunt, Seong-Kyun Lee, Yee Ling Lau, Osamu Kaneko, Eun-Taek Han

**Affiliations:** 1 Department of Medical Environmental Biology and Tropical Medicine, School of Medicine, Kangwon National University, Chuncheon, Gangwon-do Republic of Korea; 2 Department of Medical Research, Yangon, Myanmar; 3 Department of Parasitology, Faculty of Medicine, University of Malaya, Kuala Lumpur, Malaysia; 4 Department of Protozoology, Institute of Tropical Medicine (NEKKEN), Nagasaki University, Sakamoto, Nagasaki, Japan

## Abstract

The *Plasmodium falciparum* apical asparagine (Asn)-rich protein (AARP) is one of malarial proteins, and it has been studied as a candidate of malaria subunit vaccine. Basic characterization of PvAARP has been performed with a focus on its immunogenicity and localization. In this study, we further analyzed the immunogenicity of PvAARP, focusing on the longevity of the antibody response, cross-species immunity and invasion inhibitory activity by using the primate malaria parasite *Plasmodium knowlesi*. We found that vivax malaria patient sera retained anti-PvAARP antibodies for at least one year without re-infection. Recombinant PvAARP protein was strongly recognized by knowlesi malaria patients. Antibody raised against the *P. vivax* and *P. knowlesi* AARP N-termini reacted with the apical side of the *P. knowlesi* merozoites and inhibited erythrocyte invasion by *P. knowlesi* in a concentration-dependent manner, thereby suggesting a cross-species nature of anti-PvAARP antibody against PkAARP. These results can be explained by B cell epitopes predicted in conserved surface-exposed regions of the AARP N-terminus in both species. The long-lived anti-PvAARP antibody response, cross-reactivity, and invasion inhibitory activity of anti-PvAARP support a critical role of AARP during the erythrocyte invasion and suggest that PvAARP induces long-lived cross-species protective immunity against *P. vivax* and *P. knowlesi*.

## Introduction

*Plasmodium vivax*, the most widely distributed malaria parasite, globally contributes to 16 million cases outside of Africa, and affects the economy of most developing countries^1^. Vivax vaccine candidates have been selected on the basis of the analysis of orthologous *P. falciparum* vaccine candidate antigens^2^. Protective immune responses to the blood stage through inhibition of merozoite invasion or phagocytosis^3,4^ involve complex interactions of humoral immune responses and cell-mediated immune responses^5^. The development and maintenance of immune responses are crucial for malaria vaccine development^6^. Several studies have evaluated humoral immune responses for blood-stage antigens, including merozoite surface proteins (PvMSPs)^7^, apical membrane antigen 1 (PvAMA1), Duffy-binding protein (PvDBP)^6,7^, and tryptophan-rich antigens (PvTRAgs)^8^ of *P. vivax*. Antibody titers against the high molecular weight rhoptry protein 2 (PvRhopH2)^9^ have been found to be high, and one against rhoptry-associated membrane antigen (PvRAMA) is stably maintained^9,10^.

In addition to four well-known species of *Plasmodium* causing malaria in humans, *Plasmodium knowlesi* has been confirmed to naturally infect humans and is considered an emerging threat^11,12^. *Plasmodium knowlesi* infections are prevalent in all Southeast Asian countries, and Malaysia serves as an epicenter accounting for up to 80% of human infection^13^. Recent genetic and genomic studies have revealed at least three *P. knowlesi* sub-populations infecting humans, thus adding to the complexity of treating and managing this disease^13–15^. Because of the phylogenetically close relationship between *P. vivax* and *P. knowlesi*, drug targets and cross-species vaccine candidates for these *Plasmodium* species would be of great interest^16^. Cross-species immune response has been observed by some malarial antigens in different species^17–19^, and a robust protective response for one malaria species can be sufficiently strong to protect against multiple malaria species^19,20^. Thus, a new vaccine strategy targeting both *P. vivax* and *P. knowlesi* via cross-species immunity might be a safe and cost-effective strategy^20^.

The *P. falciparum* apical asparagine(Asn)-rich protein (PfAARP, PF3D7_0423400 in gene ID of PlasmoDB) was originally identified as a merozoite rhoptry neck protein with a predicted signal peptide sequence at its N-terminus and a size of 219 amino acids^21^. The recombinant N-terminus of PfAARP binds to erythrocytes in a trypsin- or neuraminidase-treatment sensitive manner, thus suggesting that the receptor contains protein components and sialic acids^21^. The sequence of this region is highly conserved among *P. falciparum* field isolates, and sera from endemic areas recognize this region, thus indicating that this region is immunogenic^21^. Moreover, antibodies raised against this region inhibit erythrocyte invasion by merozoites in a concentration-dependent and strain-transcending manner, thus suggesting a role for the PfAARP N-terminus during invasion and a benefit in including this region in the subunit malaria vaccine^21,22^. PfAARP has orthologs in all reported *Plasmodium* species, including the *P. vivax* apical Asn-rich protein which has been reported as PvARP from previous study, here we refer as PvAARP (PVX_090210, Sal-1 strain)^23^ and *P. knowlesi* apical Asn-rich protein (PkAARP, PKNH_0515300, H strain). Recent studies have shown that PvAARP localizes on the surfaces of merozoites with accumulation on the apical side^21,24^, in contrast to the rhoptry neck localization of PfAARP. Both PvAARP and PkAARP also contain a signal peptide sequence at their N-terminus and Asn- and proline (Pro)-rich regions toward the C-terminus. Previous studies have also shown that recombinant PvAARP protein is immunogenic in natural vivax infection^24,25^.

Because the N-terminus of PvAARP shows high homology with PkAARP, we hypothesized that this N-terminal region would be suitable for inducing cross-species protective immunity between *P. vivax* and *P. knowlesi*. Thus, in this study, we first used bioinformatics programs for B-cell epitope prediction in both species to find shared epitopes in the N-terminal region with the highest sequence identity. We then generated recombinant PvAARP and PkAARP protein to produce a specific antibody with cross-species reactivity and erythrocyte invasion inhibitory activity, by using *P. knowlesi*. Recombinant PvAARP and PkAARP proteins were recognized by sera from vivax and knowlesi malaria patients. The antibody response against PvAARP was observed for up to one year in vivax malaria patients without re-infection. These findings support a critical role of AARP during the erythrocyte invasion process by these parasites and suggest that PvAARP induces long-lived cross-species protective immunity against *P. vivax* and *P. knowlesi*.

## Results

### Structures and B-cell epitope prediction for PvAARP and PkAARP

**Figure 1:**
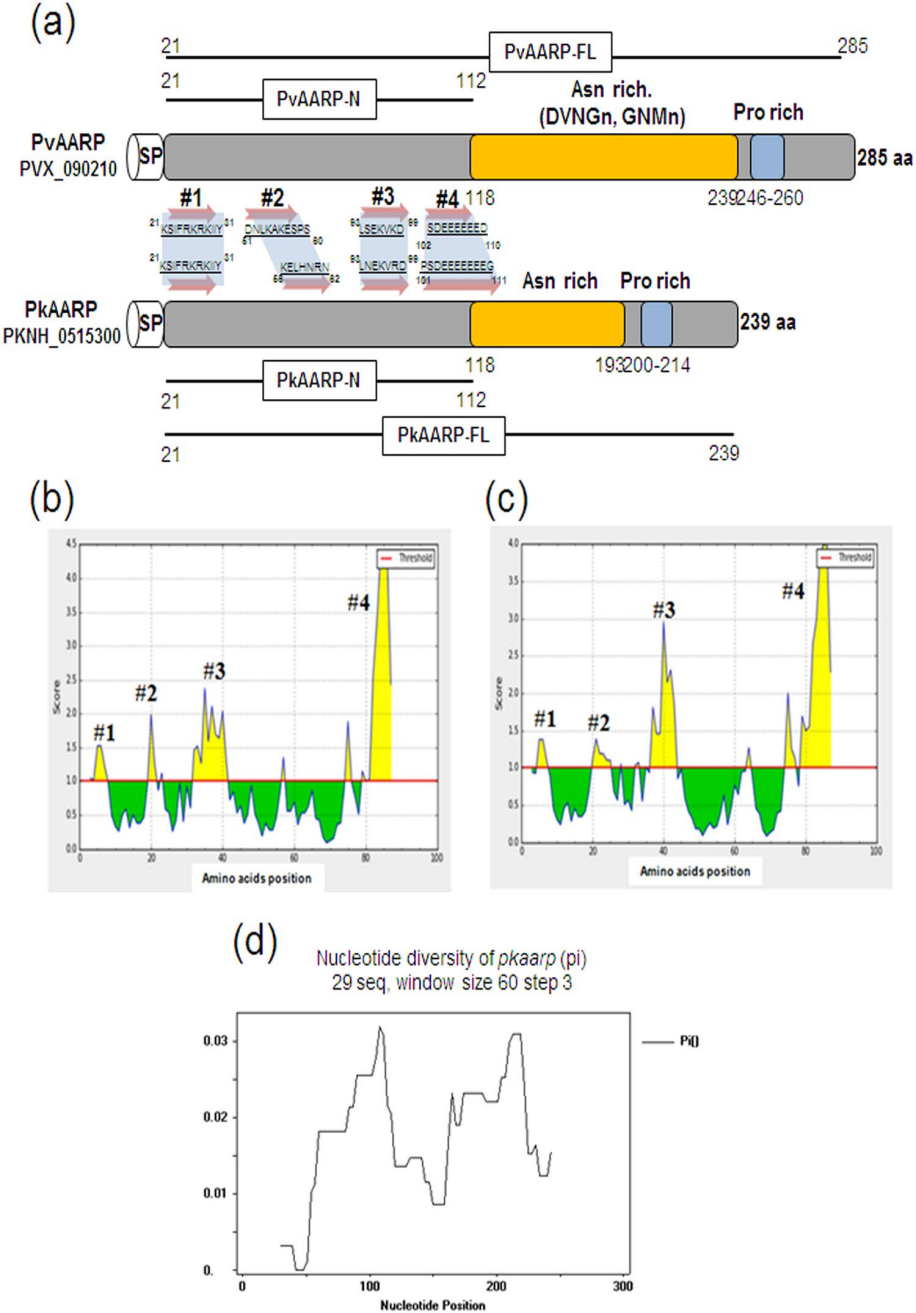
Schematic structure, B-cell epitope prediction and sequence diversity of PvAARP and PkAARP. (**a**) Schematic structures of PvAARP (285 amino acids [aa]) and PkAARP (239 aa). Asparagine (Asn)- and proline (Pro)-rich regions are indicated in yellow and blue, respectively. Regions used to generate PvAARP N-terminus (rPvAARP-N, aa 21−112), PkAARP N-terminus (rPkAARP-N, aa 21−112) and full-length PvAARP (rPvAARP-FL) and PkAARP (rPkAARP-FL) recombinant proteins are indicated under each structure. Four predicted B-cell epitopes (#1−#4) are highlighted with arrows and aa sequences. (**b** and **c**) SP, signal peptide. B-cell epitope prediction of PvAARP-N (**b**) and PkAARP-N (**c**). Yellow areas above the threshold (red line) were predicted to be part of the B-cell epitope, and green areas were unlikely to be a part of the B-cell epitope. (**d**) Sliding window plot of the nucleotide diversity of the *pkaarp* gene encoding the N-terminus using 29 *pkaarp* sequences with a window size of 60 and a step size of 3.

PvAARP (PVX_090210) and PkAARP (PKNH_0515300) contain the expected signal peptide sequence at their N-terminus and consist of 285 and 239 amino acids, respectively. No transmembrane region and GPI-anchor motif were predicted for PvAARP and PkAARP or PfAARP, according to several robust transmembrane prediction algorithms such as TMHMM ver 2.0, Phobius, and OCTOPUS^26–28^, despite a previous report of a predicted transmembrane domain in PfAARP. Thus, we inferred that AARP proteins probably do not possess a transmembrane domain. The apical Asn-rich region in PvAARP (118–239 aa) is longer than that in PkAARP (118–193 aa) (Fig. 1a). In addition to the Asn-rich region, we found that the amino acid sequences of the N-terminal region (aa 21–112 for both without the predicted signal peptide sequence) and the C-terminal region (aa 240−285 for PvAARP and 194–239 for PkAARP) of the Asn-rich region are highly conserved between PvAARP and PkAARP (74.7% and 84.8% identity, respectively). A total of four B-cell epitopes (#1−#4) were predicted for both PvAARP and PkAARP by two methods, Bcpred and IEDB web servers. The surface-exposed regions conserved between the two species were identified as the N-terminus-probable B-cell epitope regions (Fig. 1b,c). Fifty-six *pvaarp* sequences encoding the PvAARP N-terminal regions that originated from 10 countries (Brazil, China, Columbia, India, Mauritania, Mexico, North Korea, Peru, Papua New Guinea, and Thailand) were found to be 100% identical. Twenty-nine *pkaarp* sequences encoding the PkAARP N-terminal region, including 26 sequences originating from Sarawak, Malaysia^29^ and sequences from the H strain, SRA (SRS1051522), were used for the analysis (Supplementary Table S1). For 273 nucleotide positions, 20 segregating sites were found, and the nucleotide diversity was 0.01620, thus indicating that PkAARP shows limited polymorphism (Fig. 1d). Among 20 segregating sites, 9 were synonymous substitutions, and 11 were non-synonymous substitutions (Supplementary Table S2). *d*_N_ and *d*_S_ were not significantly different (Supplementary Table S3). Natural sequence variations were further tested using codon-based site-by-site analysis using FEL, IFEL, REL and MEME methods for 29 *pkaarp* sequences. A100S was identified to be under strong positive selection (Supplementary Table S3).

### Recombinant protein expression and recognition of native parasite protein

**Figure 2:**
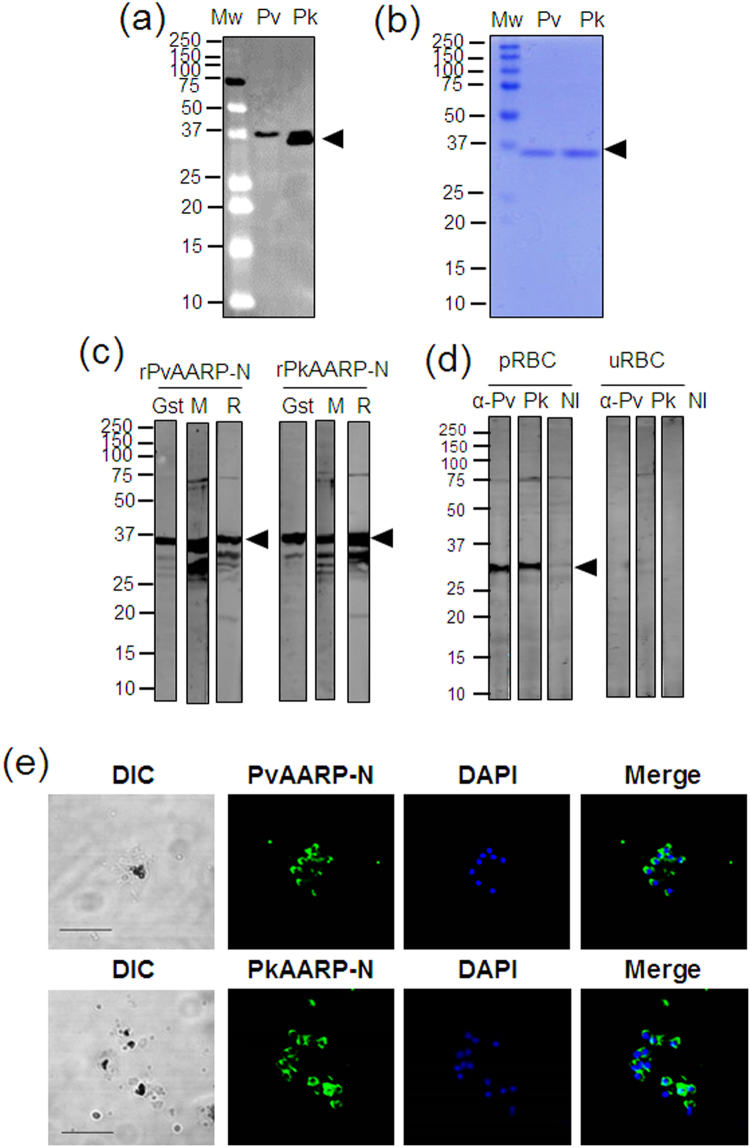
SDS-PAGE and western blot analysis of PvAARP and PkAARP. (**a**) Crude recombinant proteins of full-length PvAARP and PkAARP [rPvAARP-FL and rPkAARP-FL] were expressed with the WGCF expression system. (**b**) Recombinant PvAARP-N [Pv, 0.5 µg] and PkAARP-N [Pk, 0.5 µg] proteins were expressed in *E. coli* and purified to a single band [arrowhead]. (**c**) Specific band have been detected with anti-glutathione S-transferase antibody [Gst], mouse serum [M] and rabbit immunized [R] with PvAARP-N or PkAARP-N. (**d**) *P. knowlesi* A1-H.1 parasite lysate was recognized with anti-PvAARP-N [α-Pv] and anti-PkAARP-N [α-Pk] antibody, nor non-immunized rabbit (α-NI). The full length of SDS-PAGE and western blots are presented in the Supplementary Fig. S2. (**e**) Immunofluorescence assay of anti-PvAARP-N and PkAARP-N with *P. knowlesi* A1-H.1. pRBC, parasitized-red blood cells; uRBC, uninfected-red blood cells; DAPI, 4’,6-diaminidino-2-phenylindole. Bars indicate 5 μm.

The rPvAARP-FL and rPkAARP-FL were expressed as a 37-kDa protein by the WGCF system, and a single specific band was detected with an anti-His antibody (Fig. 2a). The recombinant PvAARP-N (rPvAARP-N) was successfully expressed and purified; it migrated as a 35-kDa single band on SDS-PAGE and was recognized by the anti-GST antibody (Fig. 2b,c, lane Gst). Anti-PvAARP-N and anti-PkAARP-N serum recognized rPvAARP-N and rPkAARP-N, respectively, as determined by western blotting analysis (Fig. 2c, lane M and R). Anti-PvAARP-N and anti-PkAARP-N antibody specifically recognized schizont parasite lysate as 35 kDa (Fig. 2d) in similar of PfAARP^21^. IFA of *P. knowlesi* with anti-PvAARP-N and anti-PkAARP-N antibody revealed that signals on merozoite surface with accumulation on one side of the merozoites (Fig. 2e, Supplementary Fig. S1A) in a similar pattern to that reported previously for PvAARP^24^.

### Cross-immunoreactivity and longevity of antibody response in patients

**Figure 3:**
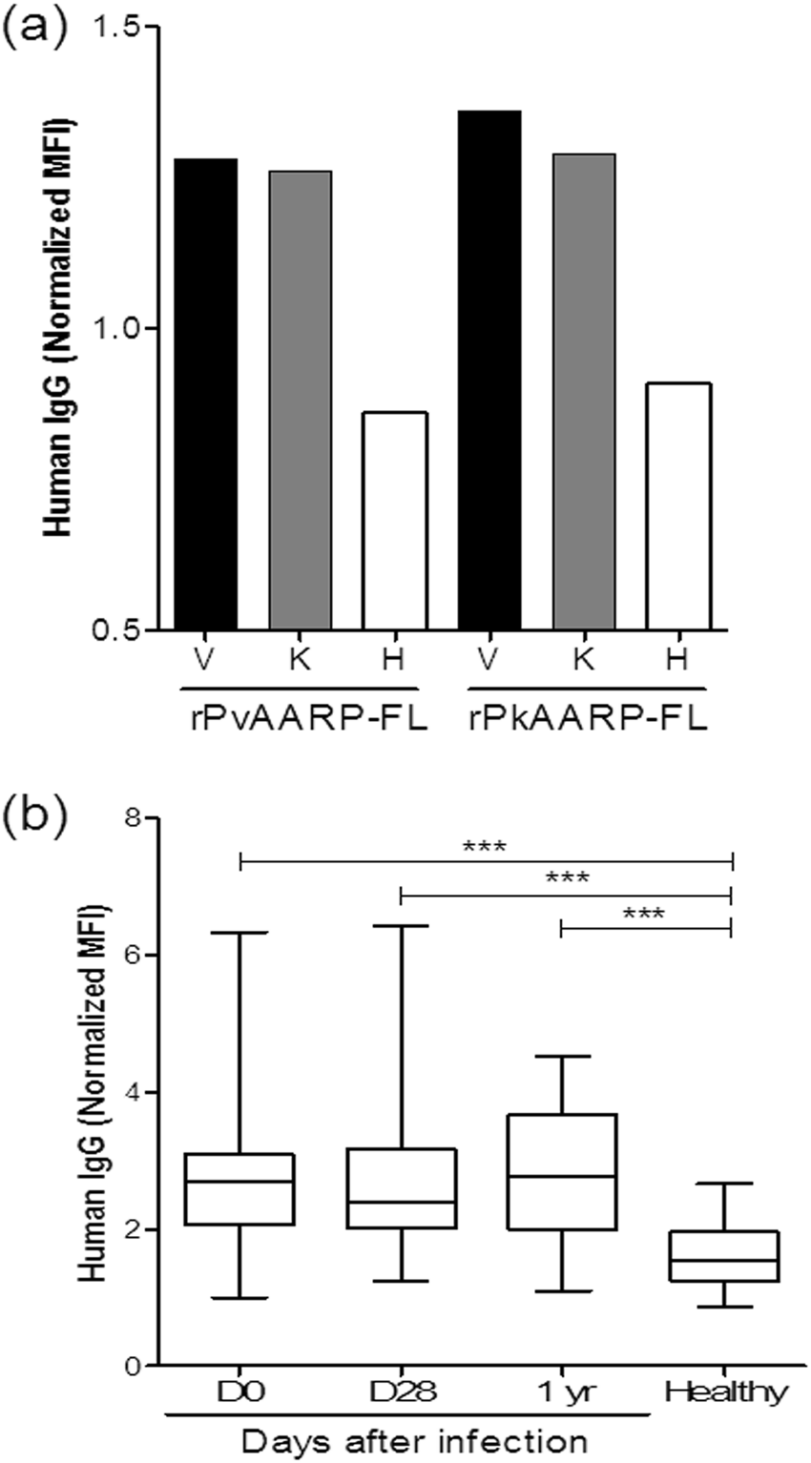
(**a**) IgG responses of pooled vivax patient serum [V], pooled knowlesi patient serum [K] or pooled healthy individual serum [H] to the full-length PvAARP-FL [rPvAARP-FL] or PkAARP [rPkAARP-FL] recombinant proteins. (**b**) IgG responses of 32 sets of archived vivax malaria patient sera to rPvAARP-FL. The IgG response is represented by normalized mean fluorescence intensity [MFI]: MFI of the test sample/[MFI + 2 standard deviations].

Protein microarray analysis revealed that both pooled vivax malaria and knowlesi malaria patient sera reacted significantly more strongly with both rPvAARP-FL and rPkAARP-FL than did sera from healthy individuals (Fig. 3a, Table 1). Notably, the reactivity of the antibody in heterologous combination was as high as that in the homologous combination. These data indicated that anti-PvAARP or PkAARP antibodies generated during the former infection probably recognize PkAARP or PvAARP in the new knowlesi or vivax infection, respectively.

**Table 1:** Characteristics of *P. vivax-* and *P. knowlesi*-infected patient samples from malaria-endemic areas.

	*P. vivax*-infected patients (Myanmar)			Pooled patient samples for cross-reactivity analysis	
	D0	D28	1 yr	*P. vivax* ^a^	*P. knowlesi* ^b^
Total (*n*)	32	32	32	8	8
Sex (M:F)	7:1	7:1	7:1	3:1	1:0
**Age (year)**					
Mean (SD)	21 (7.14)	21 (7.14)	21 (7.14)	42 (17.60)	46 (28.30)
Range	18–23	18–23	18–23	27–57	22–70
**Parasitemia (%)**					
Mean (SD)	0.40 (0.47)	0.40 (0.47)	0.40 (0.47)	0.50 (0.38)	0.18 (0.19)
Range	0.23–0.60	0.23–0.60	0.23–0.60	0.09–0.72	0.03–0.30

^a^There is no record of parasitemia level in two patients (ROK).

^b^There is no record of parasitemia level in one patient (Malaysia).

To determine the longevity of antibodies against PvAARP, 32 sets of vivax malaria patient sera from D0 (acute), D28 (sub-acute), and one year (1 yr, after infection) were evaluated (Table 1). The antibody response against PvAARP was significantly higher than that for the control healthy individuals’ sera and the response of sera collected at D0 and after 1 yr did not show significant differences (Fig. 3b, Table 2), thus suggesting that the anti-PvAARP antibody response was not diminished within one year.

**Table 2:** Seropositivity of IgG response to PvAARP in sera from human *P. vivax-*infected patients and healthy individuals.

Samples	No. of patient samples (*n*)			Normalized MFI*	95% CI^c^	No. of healthy samples (*n*)			Normalized MFI*	95% CI^c^	*p* value^d^
	Positive	Negative	Total (%)^a^			Positive	Negative	Total (%)^b^			
D0	16	16	32 (50.0)	2.75	33.6–66.4	1	31	32 (96.8)	1.62	84.3–99.5	*p* < 0.0001
D28	15	17	32 (46.9)	2.79	30.9–63.6	1	31	32 (96.8)	1.62	84.3–99.5	*p* < 0.0001
1 yr	16	16	32 (50.0)	2.76	33.6–66.4	1	31	32 (96.8)	1.62	84.3–99.5	*p* < 0.0001

*Normalized MFI: mean fluorescence intensities were divided by a cut-off value+ 2 standard deviations above the mean fluorescence intensity of the malaria-naïve samples.

^a^Seropositivity rate: percentage of positive-malaria patient samples.

^b^Seronegativity rate: percentage of malaria-naïve samples.

^c^Confidence intervals.

^d^Difference in total IgG prevalence between vivax patients and healthy individuals was calculated with Student’s *t*-test. A value of *p* < 0.05 was considered statistically significant.

### Cross-invasion inhibitory activity

**Figure 4:**
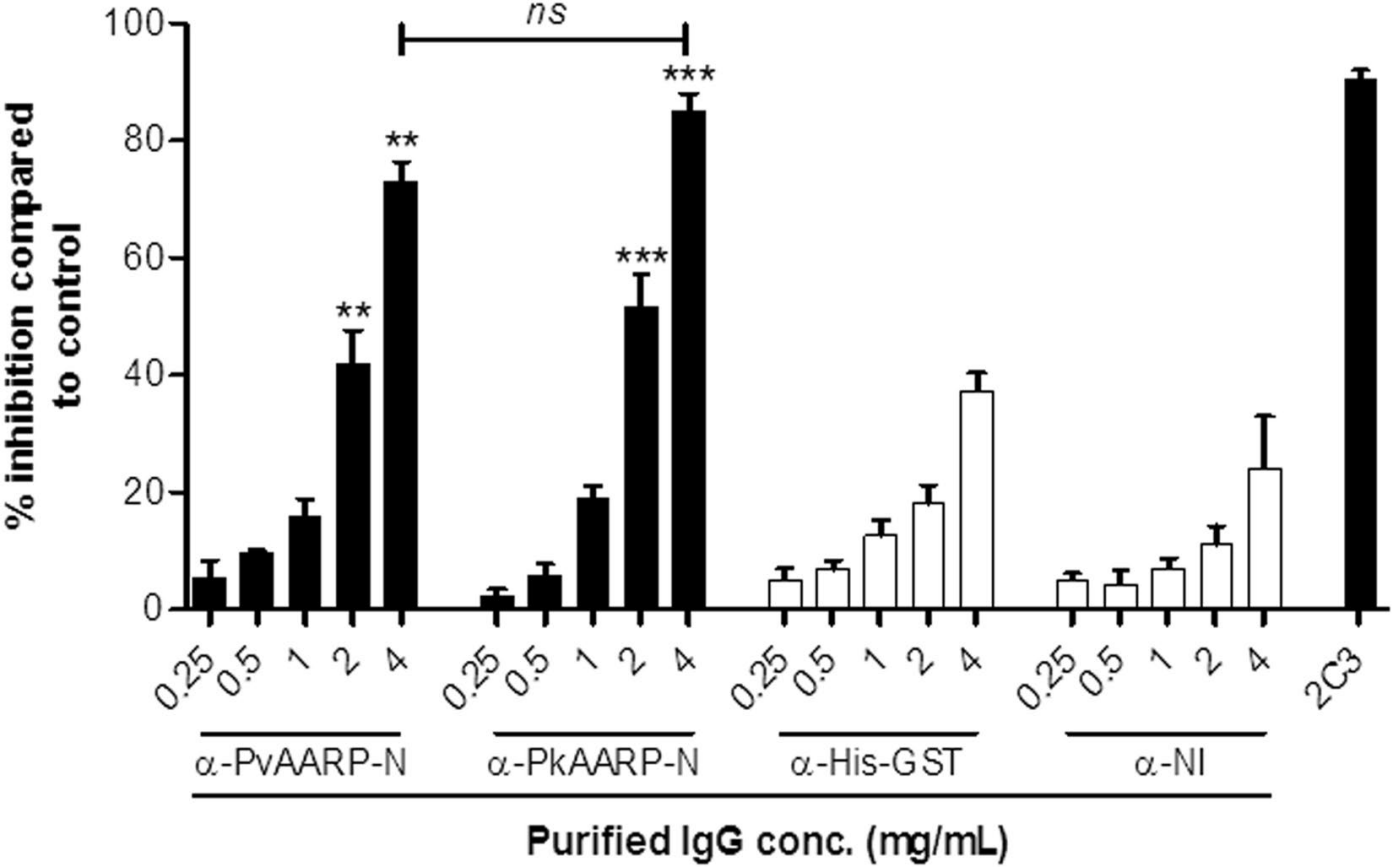
Invasion inhibition activity of anti-PvAARP-N and anti-PkAARP-N against *P. knowlesi* A1-H.1 into human erythrocytes. Purified IgG [0.25, 0.5, 1.0, 2.0 and 4.0 mg/mL] from rPvAARP-N and rPkAARP-N-immunized, non-immunized [NI], and His-GST-immunized rabbit, as well as anti-DARC [2C3] monoclonal antibodies [25 μg/mL] were examined for their inhibitory activity against erythrocyte invasion by *P. knowlesi* A1-H.1 into human erythrocytes. *ns*, not significant different. *p* > 0.05; single asterisk, *p* < 0.05; double asterisks, *p* < 0.01; triple asterisks, *p < *0.001.

To determine the cross-invasion inhibitory activity, the anti-PvAARP-N IgG antibody was administered to the *P. knowlesi* A1-H.1 and H strain culture with human and monkey erythrocytes, respectively. The efficiency of inhibition was compared to the anti-PkAARP-N antibody. We found that *P. knowlesi* invasion into human erythrocytes was significantly inhibited with purified anti-PvAARP-N IgG as efficient as anti-PkAARP-N IgG when 2 or 4 mg/mL IgG was administered (Fig. 4, no significant different (*ns*) *p* > 0.05). Whereas invasion inhibitory activity of anti-PvAARP-N antibody was found higher in *P. knowlesi* H strain at concentration <1 mg/mL (Supplementary Fig. S1B).

## Discussion

With recent advances in bioinformatics, robust algorithms have been designed and widely used to predict malaria vaccine candidates^23,30,31^. On the basis of the predictions, functional assays and antibody responses have been evaluated in *P. vivax* and *P. falciparum*^10,23,32^. One antigen is *P. falciparum* AARP, for which orthologous members have been reported in other *Plasmodium* species^21,24^. The AARP ortholog of *P. vivax* shows high homology to the primate malaria parasite *P. knowlesi*, approximately 74.7% at the N-terminal region upstream of the Asn-rich domain. Previous studies have shown that malarial antigens with high sequence homology can induce a cross-immune response^20^. We successfully expressed and purified N-terminal region of AARP in *P. vivax* and *P. knowlesi* and used for producing antibody. Indeed, we observed cross-reactivity between *P. vivax* and *P. knowlesi* via AARP proteins. The *P. vivax* and *P. knowlesi* AARP-N termini specifically recognized the merozoite surface of *P. knowlesi* with accumulation in apical end surface. The difference with PfAARP localization in apical end organelle might due to the timing of secretion. Our result by using parasite lysate also showed specific band around 35 kDa by western blot as shown in PfAARP^21^. It suggests that cross-reactivity was truly observed in AARP of *P. vivax* and *P. knowlesi* parasite level. Moreover, it might also be thought that function of AARP is conserved among *Plasmodium* spp.

In addition of cross-immunoreactivity might exist in clinical patients, immunoscreening assay was conducted. Wheat germ cell-free system was chosen for protein production as there is no glycosylation affect for incorrect immune response^33–35^. We successfully expressed the full-length domain by WGCF and unfortunately, we failed to express by *E. coli* system. The recombinant PvAARP-FL was specifically recognized by vivax- and knowlesi-infected patients. Moreover, the immunogenic strength of AARP was observed in vivax-infected patients that the anti-PvAARP could be maintained up to one year without reinfection. We also demonstrated cross-reactivity and cross-species inhibitory activity of the anti-PvAARP-N antibody against *P. knowlesi* invasion into human erythrocytes at a concentration-dependent manner, with a maximum inhibition of more than 70% when the 4 mg/mL antibody was administered. The efficiency of anti-PvAARP-N antibody to block the *P. knowlesi* invasion was as high as anti-PkAARP-N antibody. This result was consistent with the previously reported ability of the anti-PfAARP-N antibody to inhibit erythrocyte invasion by two independent *P. falciparum* strains in a concentration-dependent manner^21,36,37^. This result might prove the recognition of shared or common epitope of *P. vivax* and *P. knowlesi*. Hence, we propose that the AARP N-terminus is a putative efficacious blood stage vaccine candidate that may confer protection from both *P. vivax* and *P. knowlesi* infection in humans.

In a previous study, mice immunized with the N-terminus of PfAARP (aa 20–107) have been found to show high-titer, long-lived antibody responses for more than 4 months after immunization^38^. In the case of *P. vivax*, PvAARP has been shown to be immunogenic in most vivax patients^24^. In this study, we observed that anti-PvAARP was retained even one year after the *P. vivax* infection was resolved. In natural malaria infection, specific antibodies are often induced rapidly but also decline rapidly following parasite clearance after acute clinical malaria infection^6,32,39^. In this regard, PvAARP appears to have high potential as a vaccine candidate. B-cell epitopes predicted in the N-terminal region might induce long-lasting responses as PfAARP^38^. This finding may also suggest that memory B-cells play a role in the observed stably maintained antibody response against PvAARP in the absence of re-infection. In this study, we found that the N-terminus of *pvaarp* is completely conserved among parasites circulating in many countries in the world, and even though the *pkaarp* N-terminus contains polymorphisms, the degree of polymorphism is not high. Nonetheless, bioinformatic analyses detected one codon in the PkAARP N-terminus that was under positive selection to increase diversity, probably because of host immunity. Similar B and T cell epitope regions and natural selection have been described for other malaria antigens, such as *P. falciparum* circumsporozoite protein and apical membrane antigen proteins^40^. The PkAARP C-terminal polymorphic region was not analyzed in this study, but it may also contain codons under positive selection, and thus further investigation is needed.

In summary, we found that vivax malaria patients retained anti-PvAARP antibody responses up to one year after the infection was resolved. The cross-reactivity between *P. vivax* and the *P. knowlesi* AARP N-terminus may be due to shared B-cell epitopes identified in this study. We also showed that the anti-PvAARP antibody inhibited *P. knowlesi* erythrocyte invasion as efficient as anti-PkAARP antibody. These results support a critical role of AARP during erythrocyte invasion by these parasites and suggest that PvAARP induces long-lived cross-species protective immunity against *P. vivax* and *P. knowlesi*.

## Materials and Methods

### *P. vivax-* and *P. knowlesi-*infected patient samples

Vivax malaria patient blood samples were collected from Shwegyin Township in Myanmar on the day that the patient visited (D0, acute infection), day 28 after proper treatment (D28, sub-acute) and one year later without re-infection (1 yr). Pooled vivax-patient sera from Republic of Korea (ROK) was used after species-specific PCR of vivax-malaria was confirmed. Blood was also collected from healthy individuals living in non-endemic areas of the ROK; after malaria negativity was confirmed by microscopy and PCR; these samples were used as a negative control. Sera were separated from whole blood and used for protein microarray screening. All experiments were performed in accordance with relevant guidelines and regulations and all experimental protocols involving human samples approved by the Institutional Ethical Committee of the Department of Medical Research, Myanmar (Approval number 49/Ethics-2014) and the Kangwon National University Hospital Ethical Committee (IRB No. 2014-08-008-002). Knowlesi malaria patient sera were obtained in Malaysia between 2010 and 2013. All *P. knowlesi* and *P. vivax* patient sera chosen for this study have been confirmed by patient record files with no malaria infection history and the malaria status was also confirmed by microscopy and species-specific PCR. All experiments were performed in accordance with relevant guidelines and regulations and all experimental protocols involving human samples approved by the University of Malaya Medical Ethics Committee (Ref No. 817.18) and the Medical Research Ethic Committee (MREC), Ministry of Health, Malaysia (National Medical Research Register ID No. 13079). Informed consent was obtained from all subjects.

### *In vitro* culture of *P. knowlesi*

*P. knowlesi* H strain (a kind gift from Duraisingh MT, Harvard University)^41^ was maintained with fresh rhesus monkey erythrocytes in RPMI-1640-based medium (Invitrogen Life Technologies, Grand Island, NY) containing L-glutamine, 25 mM HEPES (Invitrogen Life Technologies) and 0.5% Albumax II (Invitrogen Life Technologies)^42^. *P. knowlesi* A1-H.1 (a kind gift from Robert W. Moon, LSHTM) was maintained with fresh human erythrocytes in RPMI-1640 in addition of human AB serum^43^. One cycle lasted approximately 28 h. Erythrocytes for *P. knowlesi* H strain *in vitro* culture were obtained from rhesus monkeys approved by the Korea National Primate Research Center, Korea Research Institute of Bioscience and Biotechnology (KRIBB) (Cheongju, Korea) and the Kangwon National University Hospital Ethical Committee (IRB No. KWNUIRB-2017-05-009-001).

### B-cell epitope prediction

The full-length *pvaarp* (PVX_090210, Sal-1 strain) and *pkaarp* (PKNH_0515300, H strain) gene sequences retrieved from PlasmoDB (www.plasmodb.org)^44^ were aligned to locate regions with high sequence identity at the nucleotide and amino-acid levels. On the basis of the highest sequence identity, the N-terminus of the PvAARP and PkAARP were chosen for B-cell epitope prediction, expression and antibody production. The *in silico* prediction of linear B-cell epitopes in the N-terminus was conducted by using the Bcpred server (http://www.imtech.res.in/raghava/bcepred/bcepred_team.html)^45^, as well as the antibody epitope prediction server at the IEDB Analysis resource, by using the Emini Surface Accessibility Prediction model (http://tools.immuneepitope.org/bcell)^46^.

### Recombinant protein expression and purification

The DNA fragment encoding the full-length *pvaarp* was amplified from a Korean vivax isolate with the primers PvAARP-FL_F (gggcggatat*ctcgag*AGTATTTTCCGAAAAAGGAAAATT) and PvAARP-FL_R (gcggtacccg*ggatcc*CTACGGCACGCCAAACAG). The DNA fragment encoding the full-length *pkaarp* was amplified from the *P. knowlesi* H strain with the primers PkAARP-FL_F (gggcggatat*ctcgag*AGTATTCTCCGAAAGAGGAAAATTATT) and PkAARP-FL_R (gcggtacccg*ggatcc*TTACGGCACGCCAAACAACTT). Small letters indicate the plasmid-derived sequence, and italicized and underlined letters indicate enzyme restriction sites, *Xho*I and *Bam*HI, respectively. PCR was run with an initial denaturation at 94 °C for 2 min, followed by 35 cycles of 94 °C for 20 sec, 60 °C for 30 sec, and 58 °C for 1 min and a final extension at 68 °C for 10 min. The amplified PCR products were purified and cloned into the pEU-E01-His-TEV-N2 plasmid vector (CellFree Sciences, Matsuyama, Japan) by using an In-fusion cloning kit (Clontech, Palo, Alto, CA) in a wheat-germ cell-free (WGCF) protein expression system. Expressed crude recombinant proteins rPvAARP-FL and rPkAARP-FL were used directly for immunoscreening by protein array.

The glutathione S-transferase (GST)-tagged PvAARP and PkAARP N-terminus (rPvAARP-N and PkAARP-N, respectively) were expressed in an *Escherichia coli* system as follows. Genomic DNA from a Korean vivax isolate was used as a template for PCR amplification with specific primer pairs PvAARP-N_F (ggatccccag*gaattc*atAAAAGTATTTTCCGAAAAAGGAAAA) and PvAARP-N_R (gatgcggccg*ctcgag*TGGCCATCCTCCTCCTCT). Genomic DNA from *P. knowlesi* H strain was used to amplify PkAARP-N with specific primers PkAARP-N_F (ggatccccag*gaattc*atAAAAGTATTCTCCGAAAGAGGAAAA) and PkAARP-N_R (gatgcggccg*ctcgag*TGACTCTCCTCCTCTTCTTCCTC). Small letters indicate the plasmid-derived sequence, and italicized and underlined letters indicate *Eco*RI and *Xho*I restriction enzyme sites, respectively. PCR was run using a high-fidelity KDO-plus Kit (Toyobo Co., Osaka, Japan) with initial denaturation at 94 °C for 2 min, followed by 35 cycles of 94 °C for 15 sec, 60 °C for 30 sec, and 58 °C for 1 min and a final extension at 68 °C for 10 min. The final amplicon was purified using a gel extraction kit and ligated into the pGEX-4T-2 expression vector (GE Healthcare, Upsala, Sweden). The obtained plasmid was confirmed by DNA sequencing analysis and transformed into *E. coli* BL21(DE3) competent cells (Invitrogen Life Technologies). GST-fused recombinant proteins were induced with 0.1 mM isopropyl-β-D-thiogalactopyranoside (IPTG; Sigma-Aldrich Co., St. Louis, MO) and purified using glutathione Sepharose 4B (GE Healthcare) according to the manufacturer’s instructions. The purity was confirmed by SDS-PAGE and western blot analysis.

### Animal antibody production

Mouse antibodies were raised against rPvAARP-N and PkAARP-N in 6- to 8-week female BALB/c mice (DBL, Seoul, Korea). Thirty micrograms of recombinant protein in PBS was intraperitoneally injected into mice with complete Freund’s adjuvant (Sigma-Aldrich Co.) in a final volume of 100 µL at first, and this was followed by two rounds of boosting with incomplete Freund’s adjuvant. Rabbit antibodies were generated in Japanese white rabbit with 250 ug purified recombinant proteins. All animal immunizations were conducted three times at two-week intervals. Sera were collected two weeks after the last boost. All animal experimental protocols were approved by the Institutional Animal Care and Use Committee of Kangwon National University and the experiments were conducted according to the Ethical Guidelines for Animal Experiments of Kangwon National University (KIACUC-16-0158).

### SDS-PAGE and western blot analysis

PkA1-H.1 schizont was lysed with tetanolysin 100 U/mL^47^ and incubated for 50 min in 37 °C. Cells were separated from supernatant and pellet. Pellet was washed with 1X complete protease inhibitor cocktail in PBS. Parasite antigen was used for western blot analysis. Recombinant proteins rPvAARP-FL, rPkAARP-FL, rPvAARP-N (0.5 µg) and rPkAARP-N (0.5 µg) were separated by 13% SDS-PAGE under reducing conditions and stained with 0.25% Coomassie brilliant blue R-250 (Sigma-Aldrich Co.). The proteins were also transferred to a 0.45-µm polyvinylidene fluoride (PVDF) membrane (Millipore, Billerica, MA) in a semi-dry transfer apparatus at a current of 360 mA for 40 min. After being blocked with 5% skim milk overnight at 4 °C, membrane-transferred proteins were reacted with primary anti-GST (1:10,000) or anti-His (1:2,000) monoclonal antibodies, animal immune sera (1:1000) and then reacted with secondary IRDye goat anti-mouse or goat anti-rabbit (1:10,000) antibodies (LI-COR Bioscience, Lincoln, NE). The Odyssey infrared imaging system (LI-COR Bioscience) and Odyssey software (LI-COR Bioscience) were used to visualize the bands.

### Immunofluorescence assay (IFA)

Parasites on the IFA slides were fixed with ice-cold acetone for 10 min and blocked with 5% BSA at 37 °C for 30 min as describe somewhere else^7^. Anti-PvAARP-N and PkAARP-N (1:50 dilution) polyclonal antiserum were used as a primary antibody and Alexa Fluor 488-conjugated anti-mouse or anti-rabbit (H + L) antibody (1:500, Invitrogen Life Technologies) was used as a secondary antibody.

### Protein microarray

Glass slides were coated with amine solution as described in a previous report^25^. Evaluation of the acquired immune response was assessed in 32 pairs of vivax sera samples (D0, D28, and 1 yr). Sera pooled from eight vivax malaria patients or eight knowlesi malaria patients were used to evaluate cross-immunoreactivity. The crude rPvAARP-FL and rPkAARP-FL proteins were spotted in duplicate, incubated at 37 °C for 2 h, blocked with 5% BSA in PBS 0.1% Tween-20, and incubated at 37 °C for 1 h. The rPvAARP-FL-coated slides were reacted with sera from malaria patients or healthy individuals (1:25 dilution) at 37 °C for 1 h. Alexa Fluor 546-conjugated goat anti-human IgG (10 µg/mL; Invitrogen Life Technologies) was used as a secondary antibody, and the fluorescence signal was detected with a scanner (InnoScan scanner, Carbonne, France). The cut-off values were set to the mean fluorescence intensity (MFI) plus 2 standard deviations (SDs). The normalized MFI was calculated by dividing the MFI by the cut-off value.

### Invasion inhibition assay

*P. knowlesi* schizonts were purified by using MACS technology (Miltenyi Biotec, Bergisch Gladbach, Germany) and cultured in 96-well plates in a total 100-µL volume in each well. Hematocrit and initial parasitemia were adjusted to 2% and 1.0–1.5%, respectively. Purified anti-PvAARP-N and PkAARP-N IgG antibodies at different concentrations were added to the 96 well plate (test wells). Control wells without antibody (normal invasion well) were set to validate the normal invasion ability of the parasite. The culture was incubated at 37 °C in a humidified culture chamber for approximately 10 h until newly invaded ring stage parasites were found. Each assay was performed independently in duplicate thrice. The non-immunized mouse or rabbit IgG, anti-His-GST IgG, and anti-Fy6 monoclonal antibody against Duffy antigen receptor for chemokines (DARC) (2C3; 25 μg/mL; a kind gift from Renia L, Singapore Immunology Network-BMSI-A STAR)^48^ were used as a baseline control. The parasites were stained with SYBR Green I (Sigma-Aldrich Co.). Briefly, cultures were centrifuged at 500× g for 5 min, and pellets were washed two times with filtered 1× PBS and fixed with 0.05% glutaraldehyde (Sigma-Aldrich Co.) for 10 min. Fixed samples were washed twice, stained with SYBR Green I at 0.2× dilution for 10 min, and washed twice. The samples were analyzed with an Accuri C6 flow cytometer (Accuri cytometers Inc., Ann Arbor, MI). In total, 200,000 cells were recorded. Percent inhibition was calculated using the following formula: 100 – (100× (test well/normal invasion well)).

### Genetic analysis of N-terminal region of *pvaarp* and *pkaarp*

*Pvaarp* sequences were obtained through PlasmoDB^44^. All raw sequences were analyzed and trimmed using SeqMan software Lasergene ver. 7.0 (DNASTAR Co., Madison, WI) to contain nucleotide positions 61–333, based on the Sal-1 *pvaarp* sequence. Sequences were aligned using CLUSTAL-W in MegAlign, Lasergene ver. 7.0 (DNASTAR Co.). Nucleotide diversity (π), defined as the average number of nucleotide differences per site between the two sequences, and the numbers of polymorphic sites (S) and haplotypes (H) were calculated using DnaSP ver. 5.0^49^. Non-synonymous substitutions per non-synonymous sites (*d*_N_) and synonymous substitutions per synonymous sites (*d*_S_) were compared by using a codon-based z-test implemented in MEGA ver. 7.0^50^. Natural selection acting at the N-terminal epitope region was also tested by using codon-based site-by-site analysis to detect codon sites under positive selection at the population level by determining the differences between *d*_N_ and *d*_S_ per site using fixed-effects likelihood (FEL)^51^, internal fixed-effects likelihood (IFEL)^52^, random-effects likelihood (REL), and mixed-effects model of episodic selection (MEME) methods^53^.

### Statistical analysis

Data analysis was performed using GraphPad Prism (GraphPad Software, San Diego, CA). The student’s *t-*test was used to compare mean from two groups or a one-way ANOVA with Tukey post hoc test was used to evaluate the significant differences between the means from more than two groups. The values of *p* < 0.05 were considered significantly different.

## Electronic supplementary material

Supplementary Figures

Supplementary Tables
