# Supplementary material for: Cross-species analysis of apical asparagine-rich protein of Plasmodium vivax and Plasmodium knowlesi
Source: Sci Rep. 2018 Apr 10;8:5781. doi: 10.1038/s41598-018-23728-1 (PMC5893618; doi:10.1038/s41598-018-23728-1)
Supplement: Supplementary file 1 — Supplementary Figures [file 41598_2018_23728_MOESM1_ESM.pdf]

Supplementary information

Title

Cross-species analysis of apical asparagine-rich protein of *Plasmodium vivax* and *Plasmodium knowlesi*

Author names and affiliations

Fauzi Muh[1], Md Atique Ahmed[1], Jin-Hee Han[1], Myat Htut Nyunt[1,2], Seong-Kyun Lee[1], Yee Ling Lau[3], Osamu Kaneko[4], Eun-Taek Han[1,*]

Supplementary Fig. 1

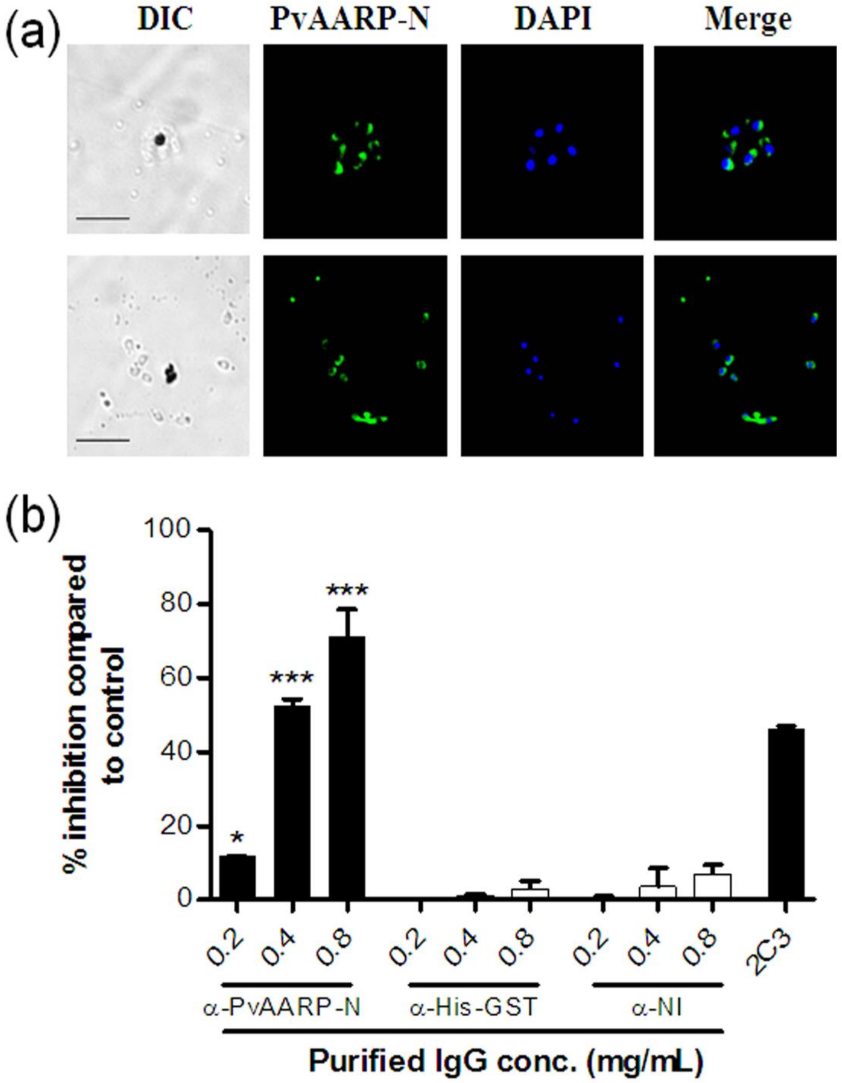

Supplementary Fig. S1 Immunoflurescence (A) and invasion inhibition activity of anti-PvAARP-N in *P. knowlesi* H strain into monkey erythrocytes (B). Significant different *, p value < 0.05;**, p value < 0.01; ***, p value < 0.001 compared to anti-His-GST. DAPI, 4’,6-diaminidino-2-phenylindole. Bars indicate 5 μm.

Supplementary Fig. 2

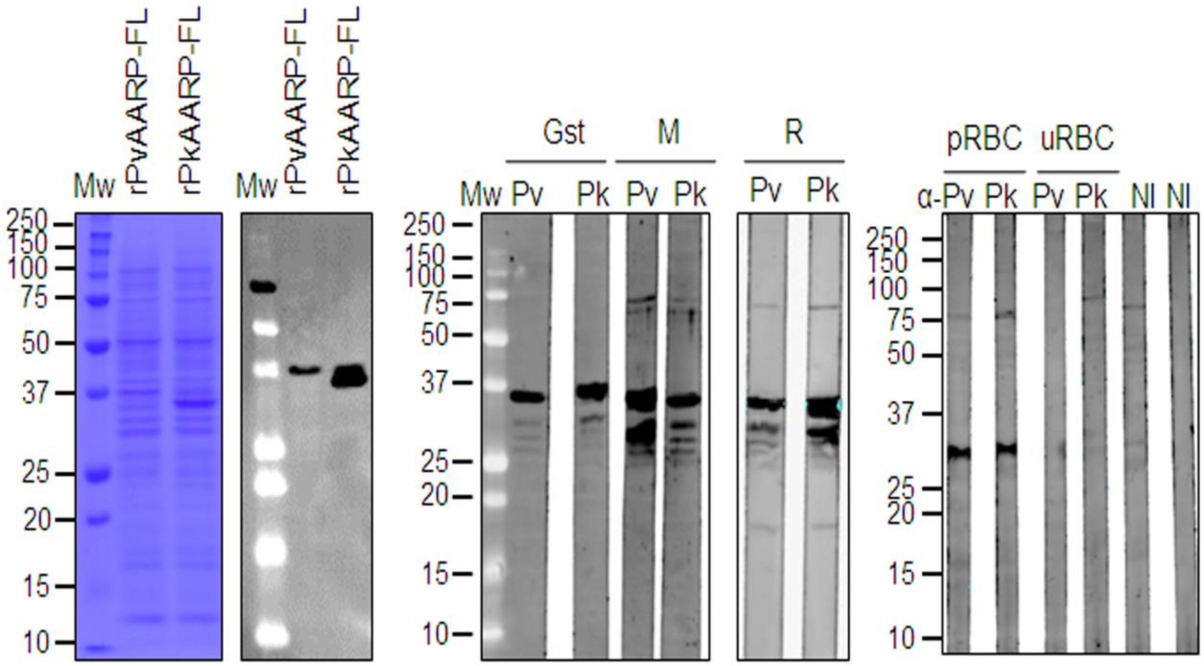

Supplementary Fig. S2 Full length of SDS-PAGE and western blots analysis in Figure 2A-D. Pv, rPvAARP-N; Pk, rPkAARP-N, Gst, anti-GST antibody; M, mice antibody, R, rabbit antibody; NI, non-immunized antibody; pRBC, parasitized-red blood cells; uRBC, uninfected-red blood cells.
